# Cognitive and Motor Development in Autistic Children: A Multi-Modal Feasibility Study Combining Neuroimaging and Virtual Reality Paradigms

**DOI:** 10.3390/brainsci16090947

**Published:** 2026-09-05

**Authors:** Maria J. Ayoub, Aarani Kamalanathan, Abigail Frankenberg, John Mutersbaugh, Minh Vu, Tanya S. Azar, Thien Nguyen, Amir Gandjbakhche, Alan H. Gerber

**Affiliations:** 1 Eunice Kennedy Shriver National Institute of Child Health and Human Development, Bethesda, MD 20894, USA; ayoubmj@nih.gov (M.J.A.);; 2 Children’s National Hospital Center for Autism, Shady Grove Office, Rockville, MD 20850, USA

**Keywords:** autism, pediatrics, functional near-infrared spectroscopy, virtual reality, motor development and performance

## Abstract

**Highlights:**

**What are the main findings?**

- The use of virtual reality will produce substantial kinematic data of upper limb motor performance, including reaction time, movement time, time to peak velocity and acceleration, and normalized jerk.
- The neural mechanisms underlying these movements, assessed via functional near-infrared spectroscopy, will provide a deeper, neurobiological context for these kinematic data.

**What are the implications of the main findings?**

- Multimodal methods incorporated into motor development and performance research are essential to establishing a comprehensive understanding of clinical populations with differences across multiple developmental domains.
- Combining kinematic data with neural biomarkers will lay the foundation for supplementing behavioral assessment methods in autism, as well as contribute to potential cognitive and motor intervention and support strategies using virtual reality.

**Abstract:**

**Background/Objectives**: In both typical and atypical development, there is growing evidence that motor development sets the stage for development in other domains. This study aims to examine the feasibility of conducting cognitive and motor research-based tasks in a virtual reality (VR) environment with concurrent neuroimaging (functional near-infrared spectroscopy, or fNIRS) in autistic children. **Methods**: During a single study visit, participants will complete research-based tasks conducted in a virtual reality environment while wearing the fNIRS apparatus. Supplementary procedures will include a range of standardized assessments and questionnaires for caregivers and their children to complete. **Anticipated Outcomes**: These methods will yield a rich combination of data -- the kinematic data associated with performance during the research-based tasks, as well as the neural correlates underlying this performance. **Conclusions**: The overarching, long-term goal of this feasibility study is to explore the interrelationships between cognitive, motor, and social development in autistic children using a variety of research methodologies. A better understanding of these relationships and their neural correlates will inform future research, assessment methods, and intervention strategies across the autism spectrum.

## 1. Introduction

Foundational research in motor development suggests that motor development is a lifelong process, with significant periods and stages occurring throughout childhood and adolescence [1]. Most fundamental patterns of movement are well-established by the age of 7—however, beyond this point, motor development remains a continuous and adaptive process. For example, school-age children between the ages of 8–12, situated within the “context-specific” period of motor development, are learning to apply basic motor execution to motor performance within more complex settings requiring cognitive and motor skills, such as sports and games [1]. However, motor development does not simply occur alongside these changes in cognition—in both typical and atypical development, there is growing evidence that motor development sets the stage for development in other domains across the lifespan [2,3,4,5,6,7,8,9,10,11,12,13]. These complex interactions between and across developmental domains throughout the lifespan are otherwise known as developmental cascades [14]. The characteristics of the physical human body, the environment in which the body is contextualized, and the interrelations between these domains as a child grows promote opportunities for exploration, learning, and, ultimately, psychological development [14]. Hence, the study of motor development beyond infancy and across a variety of neurotypes is crucial to a comprehensive understanding of human development.

These developmental cascades, particularly with regard to motor development, remain relevant well into childhood and adolescence, with their effects becoming increasingly apparent in pediatric autism research [15]. Motor differences, which are highly prevalent among autistic individuals [16,17,18], are strongly associated with impairments across other developmental domains [19]. A wealth of literature has shown cascading effects between motor development impairments in autism and language delays [20], adaptive behavior [21,22], and communication skills [23,24]. Moreover, the risk of motor impairment increases with increased impairment across other areas of development [25]. Motor development research in autism has crucial implications for assessing and supporting autistic individuals both across the lifespan and across the spectrum, who have varying support needs through multiple developmental domains.

The traditional gold-standard methods of autism diagnosis and assessment rely on behavioral characteristics, particularly related to social skills and cognitive abilities. The strengths of these assessments are vast and undeniable—they are validated scientific methodologies that are rigorously tested and acknowledged as gold-standard diagnostic measures across both clinical and scientific communities. However, as with all methodologies, limitations remain—for example, the potential for clinician error or bias [26], and a tendency towards missing the female-specific manifestations of autistic traits [27]. Furthermore, commonly used standardized assessments (e.g., Autism Diagnostic Observation Schedule, Autism Diagnostic Interview-Revised) are not intended for use among those with severe motor difficulties or intellectual impairment [26,28]. Therefore, to fully understand the relationships between motor development and other developmental domains, as well as the variability within these relationships, it is crucial to utilize a comprehensive combination of methodologies and assessments. We can gain a variety of outcomes by using a multi-modal approach—in particular, combining these traditional behavioral assessments with methods that can afford various types of physical biomarkers, such as neuroimaging [29]). For example, a combination of standardized motor assessments and movement assessment via 3D motion capture or other similar technologies during research-based tasks can fully assess a child’s motor skill and motor performance via the addition of objective kinematic data [30]. Incorporating neuroimaging can provide further biomarkers of the neural correlates underlying cognitive and motor behavior. For example, a study combining fNIRS with movement tracking during a whole-body sway synchrony task found that autistic children synced less well with a social partner and swayed with smaller amplitude compared to their non-autistic peers [31]. This reduced synchrony was accompanied by lower activity in the superior temporal sulcus and inferior frontal gyrus. Interestingly, when children swayed alone rather than with the adult, the two groups moved similarly. This suggests that the differences only emerged in socially engaged conditions [31]. A related follow-up study used a “reach-to-clean-up” task that combined kinematic and fNIRS recordings across observation, execution, and synchronization conditions in autistic and non-autistic children and found that autistic children showed a greater movement overshoot, speed, and movement segmentation both when moving alone and when synchronizing with an adult, along with reduced prefrontal and temporal activation and higher frontoparietal activation compared to the non-autistic group [32]. fNIRS has also been used with movement-based interventions; in fact, a preliminary randomized control trial used fNIRS to measure cortical activation during a drumming synchrony task before and after eight weeks of creative movement, general movement, or sedentary play interventions in children with ASD, highlighting how fNIRS can serve not only as an assessment tool but as an outcome measure [33]. Together, these studies show that combining brain imaging with movement tracking is a feasible approach for understanding motor and neural profiles of autistic youth and linking brain activity to both movement patterns and intervention response. Even so, this work has stayed narrowly focused on synchrony-based tasks or more specific tasks rather than broader everyday motor skills that are important to target in autistic children. Virtual reality (VR) is another emerging technology being used to assess motor ability [34,35] and conduct motor interventions in autistic youth [36,37]. The overarching work in this growing field, primarily intervention-based, suggests that VR-based interventions are likely feasible and effective in improving motor skills in autistic children [38]. VR has also been successfully used in interventions targeting other developmental domains in autism, such as social and communication skills [39,40,41]. Pairing a custom VR game with low-cost motion tracking, Hocking et al. had ten children and adolescents with autism complete six 20 min VR sessions over two weeks to target their gross motor skills. The authors note that manual motor difficulties in ASD predict adaptive behavior up to 12 years later, underscoring why early motor interventions matter [36]. Another pilot study offers early but meaningful insight into how children and adults with ASD use vision and proprioception in immersive VR. Notably, every participant, including those with higher support needs, readily accepted wearing the head mounted display [42]. Incorporating VR in motor assessment provides real-time measures of human movement and affords movement assessment during a diverse array of research-based VR tasks. These elements are crucial to supplementing standardized movement assessments and producing comprehensive motor profiles in clinical populations.

While much work has been conducted using these assessment techniques individually, the body of research combining these uniquely informative measures of motor, cognitive, and social development remains limited. Few studies combine VR-based tasks and interventions with standardized assessments. Many VR-based studies also lack a deeper understanding of the neural mechanisms underlying development in these domains; however, the use of neuroimaging has been a valuable supplement in previous studies involving more traditional research-based tasks [27]. In particular, neuroimaging methods that can be used during human movement and collect real-time data during task performance are of great value. A prime example is functional near-infrared spectroscopy (fNIRS), which uses near-infrared light to measure brain activity indexed via changes in blood oxygenation concentration. Although it provides a regionally limited hemodynamic measure, fNIRS offers good spatial resolution and is noninvasive, portable, suitable for research involving movement, and relatively well-tolerated by autistic children [43]. In comparison to other neuroimaging techniques such as functional magnetic resonance imaging (fMRI), fNIRS provides a measure of neural activity with superior temporal resolution in milliseconds in comparison to fMRI which lags neural activity due to the temporal resolution being restricted by hemodynamic response [44]. Although fNIRS does have restricted ability to localize brain activity while fMRI is able to provide whole brain coverage, fNIRS is more attractive to this population because of its accessibility, portability, and low cost [44]. While magnetoencephalography (MEG) provides high temporal resolution images of the brain’s electrophysiological images, it is restrictive to movement and hemodynamic changes in comparison to fNIRS [45].

While a handful of VR studies have used concurrent fNIRS as a means of understanding the neural mechanisms underpinning task performance during movement (e.g., [46,47,48]), it remains a relatively limited field, particularly in research involving neurodiverse participants. Still, work outside the motor domain hints at what this pairing could offer. Bulgarelli et al. combined wearable fNIRS with an immersive Cave Automatic Virtual environment to study social preference in preschoolers as children played with preferred versus assigned avatars, demonstrating that naturalistic combined VR-fNIRS assessment is feasible even in very young children [49]. Virtual reality and neuroimaging each have their own value—the former providing rich behavioral data within an easily manipulated environment and the latter providing biomarkers of these behaviors in the form of neural activity. The combination of these technologies is complementary, providing a comprehensive profile of behavior and its underlying biological mechanisms—the basis of what is needed to develop individualized and targeted therapies for patient populations as well as supplement and strengthen traditional assessment methods. Thus, further work is needed to assess the feasibility of combining neuroimaging with research-based tasks in a virtual reality environment, with neurodiverse individuals as the population of interest.

### Aims and Hypotheses

The goal of this proposed study is to assess motor performance—in relation to other developmental domains—in autistic children during research tasks in a VR environment with concurrent neuroimaging (fNIRS). In the current paper, we will focus on the cognitive and motor portions of this overall work. The groundwork for successfully executing this multi-modal study requires that we assess the feasibility of combining these methods and utilizing these tasks. In order to properly assess the feasibility of these proposed methods, we will use Orsmond and Cohn’s (2015) feasibility study objectives, with a strong focus on study and data collection procedures [50].

The overarching, long-term goal of this feasibility study is to explore the interrelationships between cognitive, motor, and social development in autistic children using a combination of research methodologies. A better understanding of these relationships and their neural correlates will inform our understanding of the autistic brain, enhance and diversify autism assessment methods with the use of neuroimaging, and contribute to intervention strategies involving VR across the autism spectrum. For this paper, our primary aim is to examine the feasibility of conducting cognitive and motor research-based tasks, specifically, in a VR environment with neuroimaging in autistic children, with a focus on preliminary kinematic data.

**Hypothesis** **1.** *Our multi-modal research approach will ultimately be tolerable and feasible across the majority of participants (as measured via objective feasibility assessment questions). The objective feasibility assessment questions focus on researcher observations during the study, such as the number of participants reaching completion of all tasks and assessments, as well as the length of completion of study per participant.*

**Hypothesis** **2.** *The majority of participants will report their VR/neuroimaging experience to be both tolerable and feasible (as measured via subjective, self-report feasibility assessment questions). The subjective feasibility assessment questions focus on the child’s feelings towards the study, such as enjoyability, and will be completed at the end of the visit.*

**Hypothesis** **3.** *Preliminary kinematic and neural data collected using these research-based tasks will be reliable and valid measurements (i.e., preliminary examination of behavioral data suggests anticipated changes in the kinematic outcome variables of interest; acceptable signal quality is attained for neuroimaging data).*

*Measures*. This study will produce kinematic and neuroimaging data for autistic youth between the ages of 8 and 12 years old. The primary outcome measures for this study include: (1) Subjective feasibility assessment (e.g., tolerability: “Was the VR headset comfortable to wear?”, “Was the brain cap comfortable to wear?”, “Did the tasks make you tired?”, “Did you feel capable of completing these tasks?”, “Did the tasks feel too challenging?” enjoyability- “Did you enjoy these tasks?”, “Would you do them again?”, “Were the tasks engaging?”). (2) Objective feasibility assessment (e.g., How long did the entire testing battery take? How many participants completed all assessments and tasks? Did the participant exhibit any behaviors that are indicative of feeling overwhelmed, overstimulated, etc.? Did the participant experience any adverse events (e.g., cybersickness)? Preliminary behavioral and neural data—how was the data quality? Are the findings reflective of previous literature?). (3) Select neural measures (e.g., fNIRS data quality indicators, such as signal to noise ratio, and oxygenated hemoglobin concentration). (4) Select cognitive and motor behavioral measures that are specific to our research-based tasks (e.g., movement time, velocity). This combination of measures will provide a comprehensive basis for assessing the feasibility of combining neuroimaging with research-based tasks in a VR environment in autistic children.

In addition to the above primary measures, further analyses of cognitive and neural outcomes (Table S1) will be performed. Participants and their caregivers will also complete a series of questionnaires and assessments evaluating cognitive, motor, and social development (Table S2). While these outcome measures (particularly those related to social development) are not the preliminary, feasibility-based aims discussed in this paper, they are an important part of the long-term goal of this work and can be found in the Supplementary Materials for reference.

## 2. Materials and Methods

### 2.1. Overview of Study Procedures

Autistic participants (i.e., individuals who already have completed diagnostic assessments and/or have a documented and verified diagnosis) will be recruited from a clinical research database through collaboration with a local Children’s Hospital. Interested participants will be screened over the phone and/or Zoom Telehealth in order to rule out exclusionary criteria and confirm inclusion criteria. Furthermore, consent and assent forms will be sent to participants’ caregivers prior to their in-person visit to read and review. The in-person consent process will occur at the visit to local Children’s Hospital.

Study staff will obtain written consent from at least one of the participant’s caregivers, and participants will be asked to read and sign or “make their mark” on an assent form. These participants will be asked specific structured questions after the study staff reviews the consent document with them, but before consenting, to ensure they have sufficient understanding to consent to the study (e.g., “what are some of things you will be asked to do in this study?” “how would you tell us if you wanted to stop being in this study?” “what are the risks of this study?”). Participants will receive a verbal explanation of the purposes, procedures, and potential risks of the study and of their rights as research participants. They will also be given the opportunity to discuss the study with caregivers prior to agreeing to participate. If it is determined that these participants cannot provide assent, they will be excluded from the study.

The investigator will explain the research study to the participant and their caregiver, and answer any questions that may arise. Researchers will emphasize to both participants and their caregivers that they retain the right to withdraw consent at any time throughout the course of the study, and that their comfort and safety is a priority. A copy of the informed consent document(s) will be given to the participant and their caregiver for their records.

After consenting at the study visit, participants will complete three research-based cognitive–motor tasks in a virtual reality environment while connected to fNIRS optodes. As previously mentioned, other participants and their caregivers will also complete a variety of questionnaires and assessments that are a part of the larger, long-term work beyond the scope of this current paper (Table S2). Further cognitive and neural outcomes, beyond those that are feasibility related, will also be evaluated (Table S1). In the following description of the methods and results, we focus on the preliminary, feasibility-based outcomes central to this paper. This protocol has been reviewed and approved by the Institutional Review Board of Children’s National Research Institute (Protocol Number: STUDY00001552).

### 2.2. Participants

This work will assess 50 autistic children and 50 non-autistic children. We anticipate having to screen a higher number of participants to accommodate ineligibility. We estimate that we will lose 10% of participants at screenings, 20% of screened participants will choose not to enroll, and 10% of participants will dropout after enrollment. Given these factors, we estimate needing to screen at least 160 participants.

All participants will be between the ages of 8 and 12 years old. This age range corresponds with the “context-specific” period of motor development, marking the transition children make from executing basic movements to incorporating these skills within more complex settings requiring cognitive and motor skills [1]. Conducting this feasibility study within this narrow yet significant age range will set the foundation for future work examining how and why autistic children might differ in their cognitive and motor abilities compared to their non-autistic peers, as well as whether these differences correspond with their engagement in complex cognitive–motor individual and group activities.

Participants will be recruited via a database in collaboration with a local children’s hospital. Eligible youth must be right-handed, between the ages of 8 and 12 years old at the time of study enrollment (inclusive), and willing to participate in the study. Child participants must be able to follow directions and answer questions as needed, with an IQ (either previously assessed within the past two years, or assessed via The Wechsler Abbreviated Scale of Intelligence—2nd Edition (WASI-II; Wechsler, 2011) during the study visit) of 70 [51]. Both the child and accompanying parent must speak English well enough to complete consent and study procedures as our staff are unable to provide assessments in any other language besides English. Autistic participants must have a prior diagnosis within the past two years and will be confirmed via the Autism Diagnostic Observation Schedule—2nd edition (ADOS-2). Participants without a clinical diagnosis should provide staff with detailed developmental history including early childhood milestones and behavior patterns. Participants will then be able to complete the ADOS-2 to receive a diagnosis. The ADOS-2 will be administered by a licensed clinical psychologist who has been previously trained in administration with a minimum of 80% inter-rater reliability. The specific modules used will depend on each participant’s level of expressive language. Non-autistic participants will be confirmed to have no autism diagnosis as well as no first-degree relatives with an autism diagnosis.

Both autistic and non-autistic participants will be excluded due to: the usage of any psychiatric or neurologic medications that may affect study participation or the resulting neuroimaging data (e.g., substances affecting neurovascular coupling, including (but not limited to) certain psychostimulants [52] or antidepressants [53]; the presence of skin lesions or conditions on the head which may prevent proper application of fNIRS optodes; known developmental and intellectual disabilities, medical disabilities, or disorders (aside from autism) which would interfere with study participation (e.g., cerebral palsy, epilepsy); and uncorrected visual impairment, colorblindness, and/or deafness. Participants may also be excluded due to a BMI in the obese range which may prevent participation in fNIRS data collection [54].

### 2.3. Materials and Equipment

**Figure 1:**
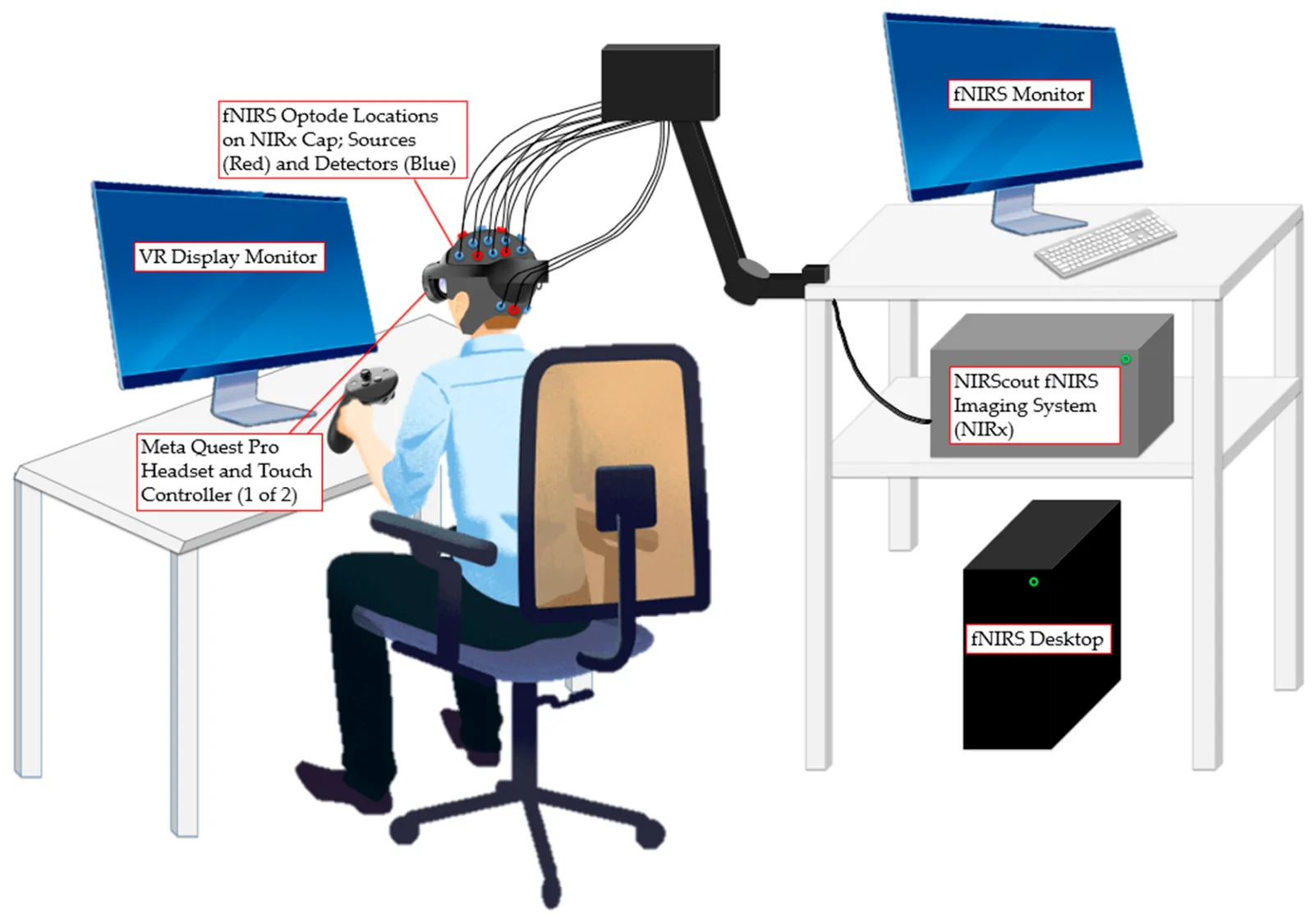
Experimental Setup. The individual, outfitted with the VR headset and the fNIRS cap and optodes, remains seated with a hand-held controller corresponding to the VR headset. The research-based tasks that they experience through the VR headset are mirrored onto an external monitor that is visible to experimenters. The desktop needed to run the devices and their synchronization are turned away from the participant and towards the experimenters. Figure created by the authors using Canva (Canva Pty Ltd., Sydney, Australia. Canva.com; accessed 8 July 2026).

**Figure 2:**
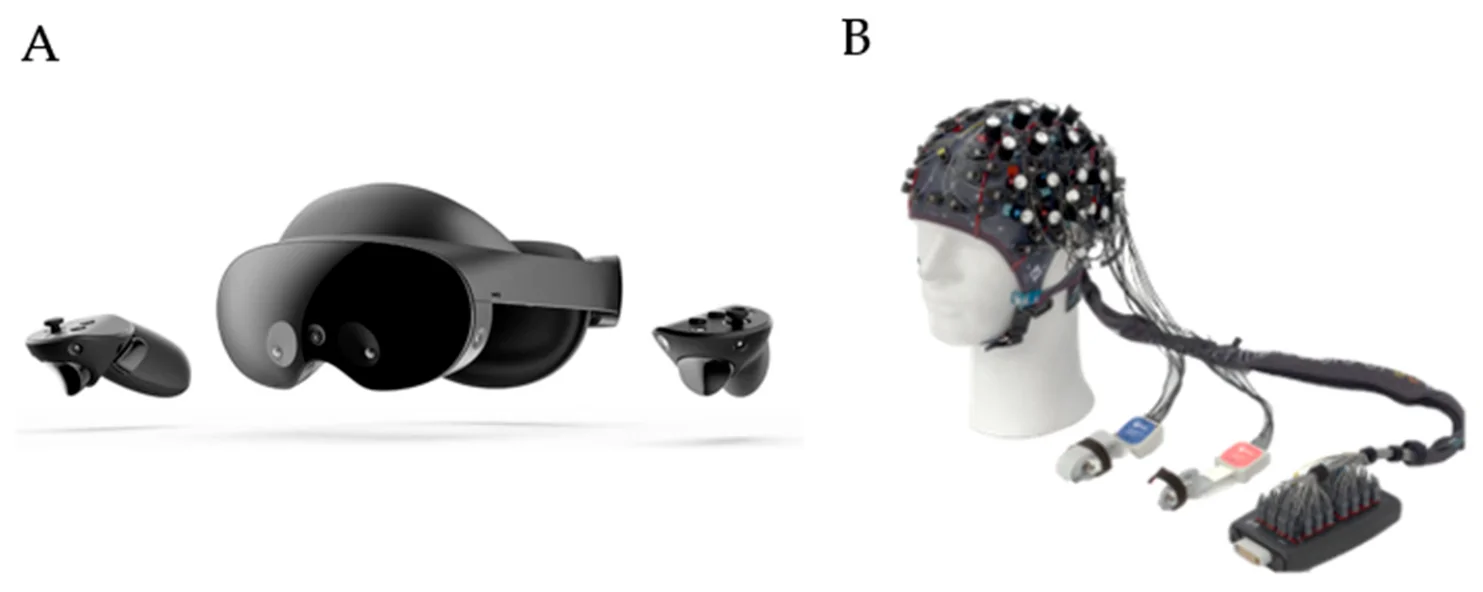
Materials and their specifications. (**A**) Meta Quest Pro Headset (722 g) and Touch Controllers (215 mm × 142 mm × 95 mm). (**B**) NIRx cap with fNIRS optodes attached (visually depicted as red sources and blue detectors in Figure 1). The NIRx NIRScout device, a continuous wave system, will collect data via 8 LED sources and 8 detectors at a sampling rate of 10 Hz.

We will use the Meta Quest Pro Virtual Reality Headset (Meta Platforms, Inc., Menlo Park, CA, USA; released 2022) and Touch Controllers (Meta Platforms, Inc., Menlo Park, CA, USA) to collect kinematic data. All the VR task development and code will be written using Unity (Version 6000.4.7f1, Unity Technologies, San Francisco, CA, USA), and all the tasks are designed to be fully immersive. The tasks will be designed for use by right-handed individuals specifically. The Meta Quest Pro is a standalone headset with integrated eye and face tracking via 10 onboard sensors, dual LCD displays at 1800 × 1920 pixels per eye, and a 106° × 96° field of view. The controller sampling rate is 1000 Hz, and the Unity frame Refresh Rate is 90 Hz. Our neuroimaging method will involve use of the NIRScout fNIRS system (NIRx Medical Technologies, Glen Head, NY, USA) and the NIRStar (v15.3) data acquisition software, sampling at a rate of 10 Hz. Figure 1 depicts the full experimental setup, with specific materials and their specifications highlighted in Figure 2.

#### fNIRS Montage Design

**Figure 3:**
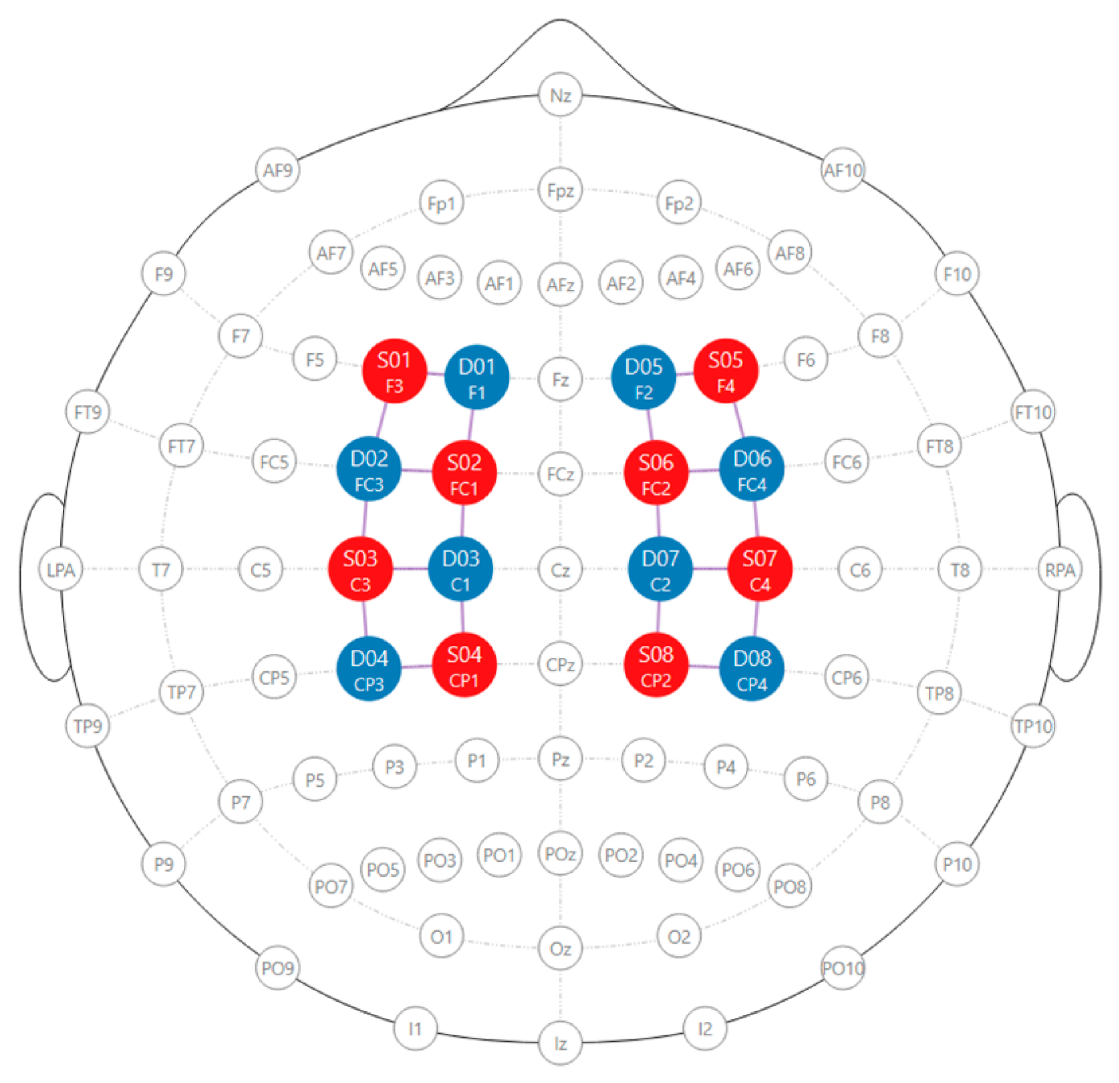
Optode layout across frontal and motor areas of interest, consisting of 8 sources (red) and 8 detectors (blue).

The fNIRS probe design follows the 10/10 electrode placement system and will be designed in NIRSite (Version 2023.9; NIRx Medical Technologies, Berlin, Germany). The design, while relatively limited due to the placement of the VR headset, will involve 8 sources and 8 detectors (20 channels), centered bilaterally across frontal and motor areas. The interoptode distance will remain standard (30 mm). Figure 3 below depicts a proposed montage, designed in NIRSite, with channels overlying these brain regions of interest.

### 2.4. Data Synchronization

Synchronization between VR events and fNIRS data will be achieved using Lab Streaming Layer (LSL), a technique commonly used for temporal synchronization in multimodal research [55]. First, during the initialization of the VR task, the headset will open a TCP/UDP socket to a relay laptop on the same local network, establishing a connection to a relay laptop via a custom Python (v3.13.15) code. The relay laptop, running a Python LSL script, then accepts the connection from the headset and ultimately creates an outlet for VR event markers and an inlet to receive fNIRS data. The Python script locates the fNIRS data stream and continuously pulls the samples with precise timestamps of triggered events, such as task start and user response times. These events are sent from the VR headset to the relay laptop, with their corresponding time stamps marked in the fNIRS time domain.

To note, LSL uses a network time protocol (NTP)-inspired clock offset measurement technique to measure the difference between the outlet and inlet clocks and adjust the delay. This correction is done periodically and utilizes a recursive least squares filter to reduce jitter effects. This allows the VR events and fNIRS measurements to be aligned within millisecond-level accuracy to streamline further data analysis.

### 2.5. Research-Based Task Design and Protocol Overview

Our cognitive–motor research-based task involves three variations of a balloon popping task, adapted from a bubble popping task used in Minissi et al., 2023 [34]. These require participants to reach and click, or “pop,” a series of balloons via their VR controller. The balloons presented as targets for participants vary in color according to the task. Conceptually, the “balloon-popping” concept was designed around the foundation of Fitts’s Law, which emphasizes a speed/accuracy tradeoff while reaching between targets [56]. In the motor control and performance literature that incorporates Fitts’s Law into the task design, the difficulty of target-reaching tasks is often manipulated by changing the size of the targets themselves or the distance between them. For the sake of relative simplicity in our study design, we chose to keep the motor difficulty constant across our task conditions—the size of the balloons remains the same throughout, and the initial “target” is the participant’s “reset” position, where they bring their hand to rest under their chin after each trial.

**Figure 4:**
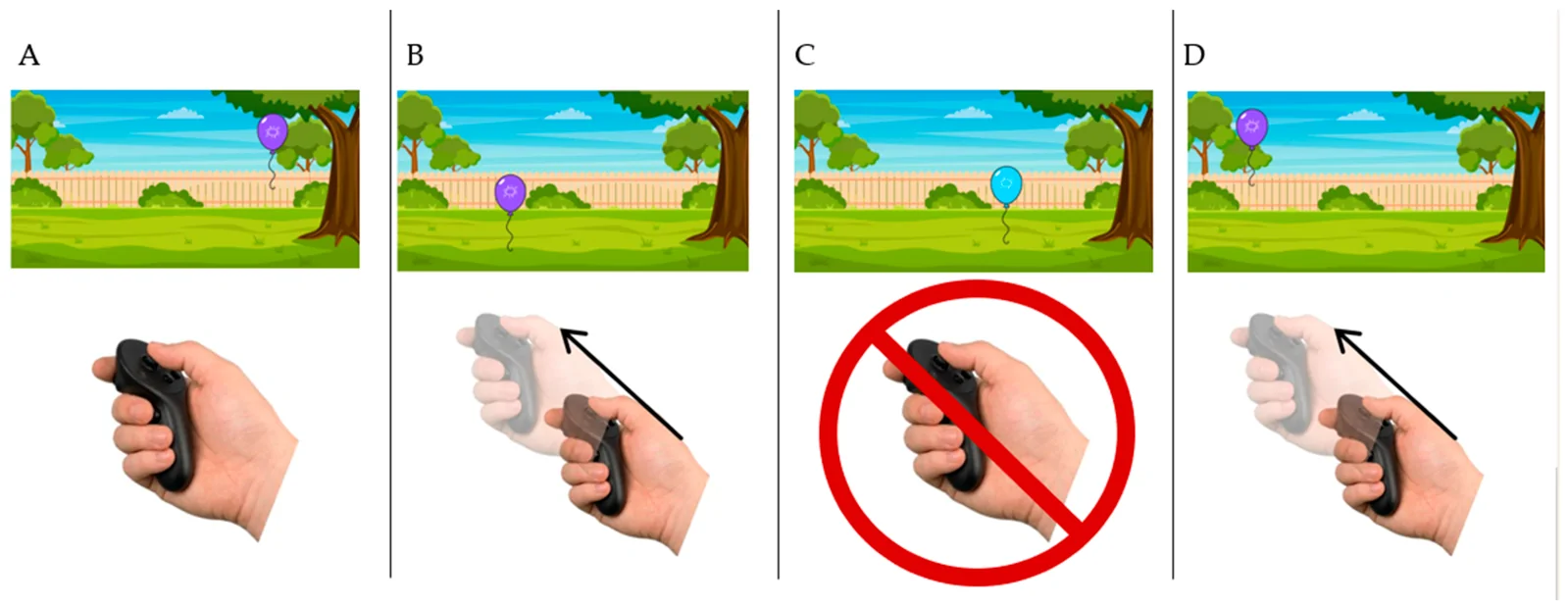
Virtual reality tasks. In each Figure above (**A**–**D**), the top image reflects the VR environment that the participant will see via the VR headset and corresponds with the task descriptions for each condition described above in Table 1. The bottom image in each figure represents the action required of the participant in each respective condition. (**A**) The “Go/No-Go” Task (cognitive condition), in which participants will simply click a button on the controller to pop the balloons that appear. (**B**) The “Reach and Pop” Task (motor condition), in which the participant will reach towards the balloon’s location and then pop the balloon using the button press on the controller. (**C**) The “Go/No-Go Reach and Pop” Task (cognitive–motor condition), with a “No-Go” trial displayed—because the balloon is not purple, the participant must inhibit their response and not reach towards or pop the balloon. (**D**) The “Go/No-Go Reach and Pop” Task (cognitive–motor condition), with a “Go” trial displayed—because the balloon is purple, the participant is expected to reach towards the balloon’s location and then pop the balloon using the button press on the controller. Figure created by the authors using Canva (Canva Pty Ltd., Sydney, Australia. Canva.com; Accessed 8 July 2026).

**Figure 5:**
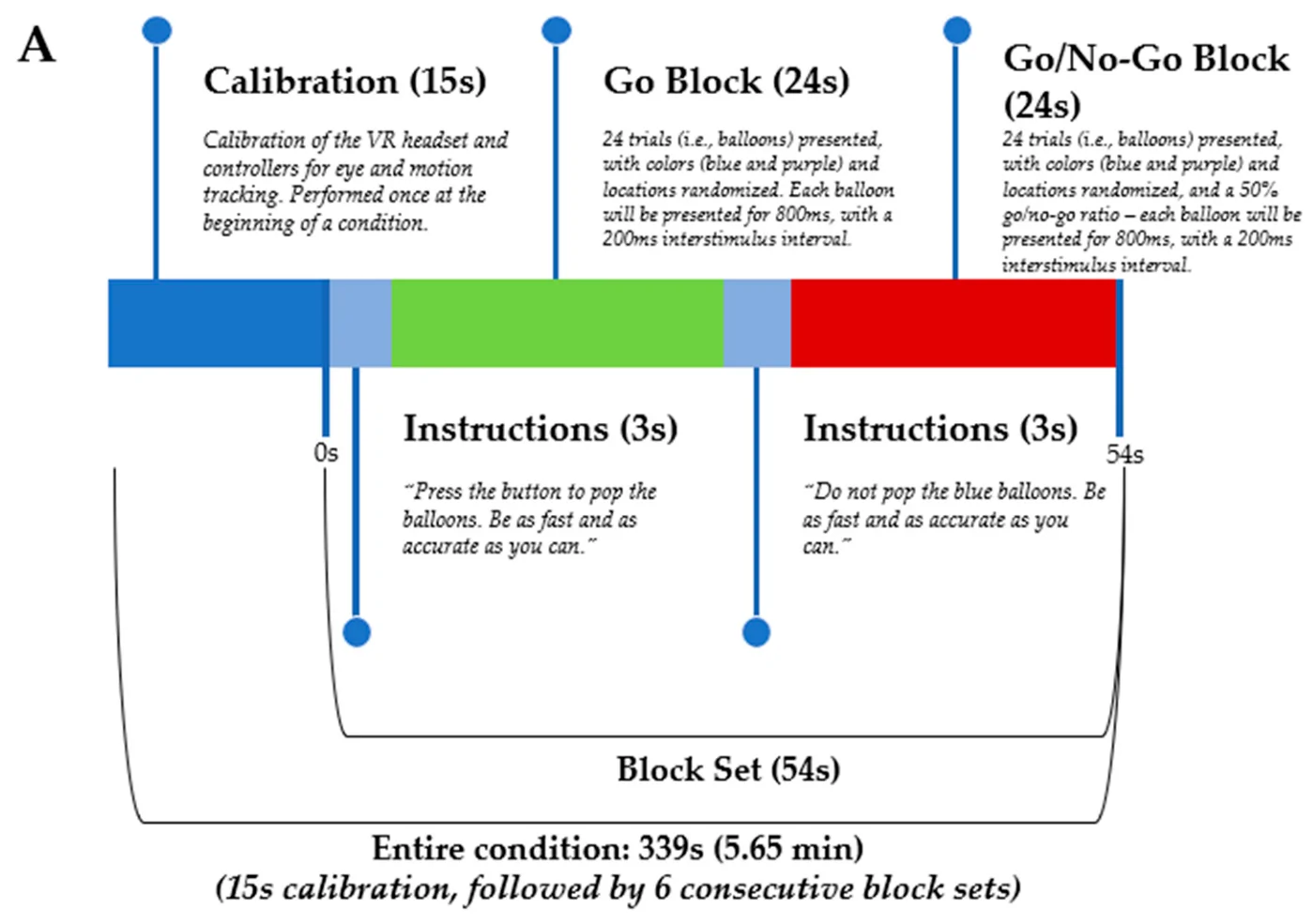

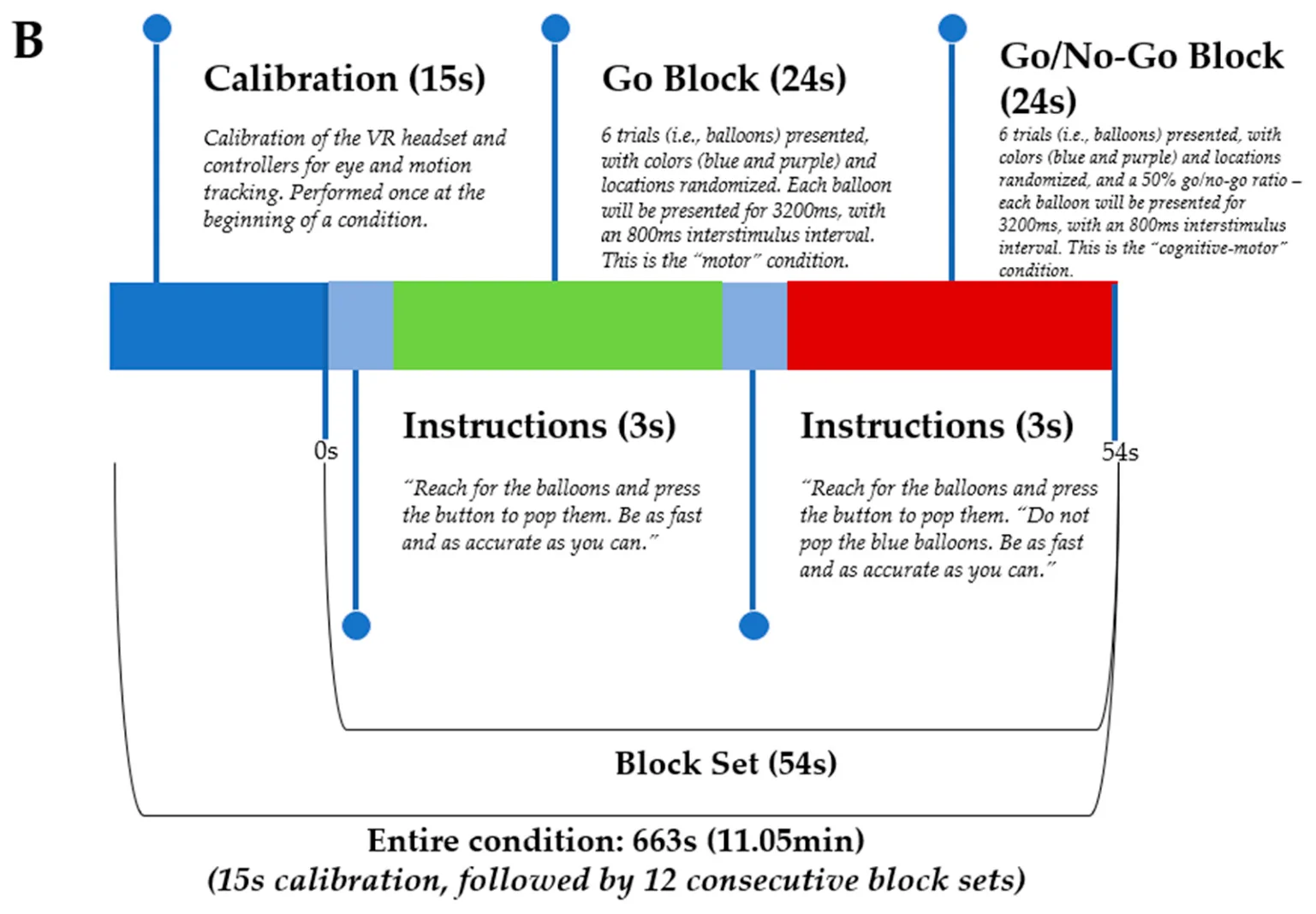
Block design breakdown. There will be two task conditions: cognitive, during which no reaching movements are involved (**A**), and motor/cognitive–motor (**B**). (**A**): The cognitive condition will consist of 6 block sets, with each task block (either go or Go/No-Go blocks) running for 24 s. Each task block will also be preceded by 3 s of brief instructions (e.g., “Press the button to pop the balloons. Be as fast and as accurate as you can.”). Therefore, one block set (two 24 s blocks, with two sets of 3 s instructions) will total 54 s, with 6 consecutive block sets totaling 5.4 min (or 324 s). Prior to the condition, there will also be a 15 s calibration period for the headset and controllers, totaling 339 s (5.65 min) for the cognitive condition. (**B**): The motor/cognitive–motor condition will consist of 12 block sets, with each task block (either go or Go/No-Go blocks) running for 24 s. Each task block will also be preceded by 3 s of brief instructions (e.g., “Press the button to pop the balloons. Be as fast and as accurate as you can.”). Therefore, one block set (two 24 s blocks, with two sets of 3 s instructions) will total 54 s, with 12 consecutive block sets totaling 10.8 min (or 648 s). Prior to the condition, there will also be a 15 s calibration period for the headset and controllers, totaling 663 s (11.05 min) for the motor/cognitive–motor condition.

To manipulate cognitive difficulty of the balloon popping tasks, we incorporated the “Go/No-Go” paradigm by changing the color of the balloons presented to participants and asking them to only pop certain colors. The traditional Go/No-Go paradigm is designed to test response inhibition—for example, a participant may be asked to refrain from responding to a stimulus of a particular color. We incorporated this paradigm to manipulate the cognitive difficulty of our balloon-popping task across three conditions: Go/No-Go, Reach-and-Pop, and Go/No-Go Reach-and-Pop. For example, in the Go/No-Go condition, participants must only pop purple balloons when they appear, ignoring all other balloon colors presented. Respectively, the Go/No-Go, Reach-and-Pop, and Go/No-Go Reach-and-Pop tasks are intended to measure cognitive, motor, and cognitive–motor domains. We will assess a small selection of cognitive outcomes of the Go/No-Go task, including commission errors (i.e., an erroneous response to a No-Go signal during a Go/No-Go trial) and omission errors (i.e., the failure to respond to a Go signal during a Go/No-Go trial). See Table 1 for full descriptions of each task condition and their respective cognitive and motor outcomes, Figure 4 for visual depictions of each task condition, and Figure 5 for a detailed breakdown of the timestamps embedded in the experimental block design. Table 1 also mentions the brain regions of interest for these tasks: the motor cortex for movement purposes, and the prefrontal cortex/dorsolateral prefrontal cortex for executive function purposes. fNIRS studies assessing movement [57] and those evaluating Go/No-Go performance [58] typically assess these brain regions.

### 2.6. Block Design and Task Details

The “Go/No-Go” paradigm is embedded within a block design, a common experimental design among fNIRS studies, which traditionally alternates task periods, or blocks, with periods of rest. There will be two experimental conditions: cognitive, and motor/cognitive–motor. The cognitive condition will consist of 6 block sets, with each task block (either go or Go/No-Go blocks) running for 24 s. Each task block will also be preceded by 3 s of brief instructions (e.g., “Press the button to pop the balloons”). Therefore, one block set (two 24 s blocks, with two sets of 3 s instructions) will total 54 s, with 6 consecutive block sets totaling 5.4 min (or 324 s). Prior to the condition, there will also be a 15 s calibration period for the headset and controllers, totaling 339 s (5.65 min) for the cognitive condition. The motor/cognitive–motor condition will consist of 12 block sets, with each task block (either go or Go/No-Go blocks) running for 24 s. Each task block will also be preceded by 3 s of brief instructions (e.g., “Press the button to pop the balloons. Be as fast and as accurate as you can.”). Therefore, one block set (two 24 s blocks, with two sets of 3 s instructions) will total 54 s, with 12 consecutive block sets totaling 10.8 min (or 648 s). Prior to the condition, there will also be a 15 s calibration period for the headset and controllers, totaling 663 s (11.05 min) for the motor/cognitive–motor condition. To mitigate possible carry-over effects between conditions, participants will be allotted up to five minutes to rest between the two conditions, and the order in which the conditions are presented will be counterbalanced across participants.

During the cognitive condition, each balloon stimulus will be displayed sequentially for 800 milliseconds, with an inter-stimulus interval of 200 milliseconds. In the motor/cognitive–motor condition, each balloon stimulus will be displayed for 3200 milliseconds, with an inter-stimulus interval of 800 ms. The balloons will be light blue and dark purple in the cognitive condition, and dark blue or light purple in the motor/cognitive–motor condition—in both conditions, participants will be asked to pop the purple balloons. Shades of blue and purple were specifically chosen for both conditions to control for the effect of color wavelengths on accuracy. Research has shown that shorter color wavelengths (i.e., blues and purples) are associated with higher accuracy in VR Go/No-go tasks [59]—therefore, it is plausible that including balloon color combinations with longer wavelengths in one condition versus another could have detrimental effects on accuracy that are unrelated to the nature of the condition itself. The order in which these colors appear throughout each block will be randomized for each participant, and the Go/No-Go ratio will be 50% (i.e., participants will need to respond to half the stimuli presented and inhibit their response to the other half). The locations of the stimuli will remain the same for each trial in order to control for the potential effect of randomized locations on kinematic measures.

The timing of the block design in each condition was largely based on the protocol used in a study that used fNIRS to assess Go/No-Go task performance among children with ADHD [60]. Similar to that study, our block design does not include rest periods between blocks—only one break between the two conditions—so as to not bore participants or lose their focus [60]. Furthermore, the go blocks in each condition are considered the baseline, rather than rest blocks. The study that the current design is based on suggests that, since brain activation during the Go/No-Go blocks is reflective of inhibitory control, it is more appropriate for the go blocks to be the baseline comparison, rather than rest blocks [60]. The block design timing was only altered for the motor/cognitive–motor condition to account for movement time from the hand to the balloon.

### 2.7. Participant Procedures and Instructions

Prior to each condition, each subject will perform one practice block to ensure their understanding of the task. Verbal assistance and clarification will be provided during the practice block as needed. The entire time allotted for the practice block will not exceed the total cognitive condition time (i.e., 5.65 min). If, after the maximum time allotted for practice, the participant still does not understand the task, the data collection session will be discontinued. The following instructions will be used prior the go and Go/No-Go blocks in each condition:

- Go Block Instructions (Cognitive Condition): “Press the button to pop the balloons. Be as fast and as accurate as you can.”
- Go/No-Go Block Instructions (Cognitive Condition): “Do not pop the blue balloons. Be as fast and as accurate as you can.”
- Go Block Instructions (Motor Condition): “Reach for the balloons and press the button to pop them. Be as fast and as accurate as you can.”
- Go/No-Go Block Instructions (Cognitive–Motor Condition): “Reach for the balloons, and press the button to pop them. “Do not pop the blue balloons. Be as fast and as accurate as you can.”

During the practice block, the instruction display will not have a time limit. During the actual conditions, and given that the participant is familiar with the instructions at that point in the experiment, the appropriate instructions will be displayed for 3 s prior to each block.

Signal optimization for each channel will be obtained by proper cap fit, as well as moving participants’ hair underneath optodes as needed for the near-infrared light to properly reach the scalp. This process is non-invasive and will take approximately 15 min to set up before the start of the fNIRS recording.

Throughout set-up and the research-based tasks, researchers will remain attentive to study participant fatigue and discomfort and will check in regularly to assess their comfort. If needed, researchers will adjust the length of the test sessions to each individual (e.g., a longer break between the two conditions, two testing sessions, etc.) accordingly. Furthermore, researchers will continuously emphasize that participation is voluntary and that comfort is a priority—they will immediately stop testing upon participants’ requests. Should the subject report any significant discomfort, the procedure will be immediately terminated. No clinical decisions will be based on the results of fNIRS and VR as they are both solely research paradigms.

## 3. Results and Proposed Data Analyses

The primary outcomes of the proposed study will include subjective feasibility outcomes per a self-report from the participant, as well as objective feasibility outcomes reported by the experimenter. To determine if the participant is able to tolerate the VR, we will use the Simulation Sickness Questionnaire (SSQ). The SSQ will evaluate tolerability by measuring side effects of VR such as fatigue, dizziness, and headache on a four-point scale (none, slight, moderate, severe) [36]. Further assessments of feasibility will include fNIRS signal quality, as well as preliminary measures of cognitive and motor performance. These outcomes will produce descriptive and qualitative results related to feasibility assessment. A summary of these results, including specific questions assessing feasibility, can be found in Table 2. Below are further descriptions of the neural results that will be obtained via the fNIRS apparatus and the motor performance results yielded by the VR controllers.

### 3.1. fNIRS Results

**Figure 6:**
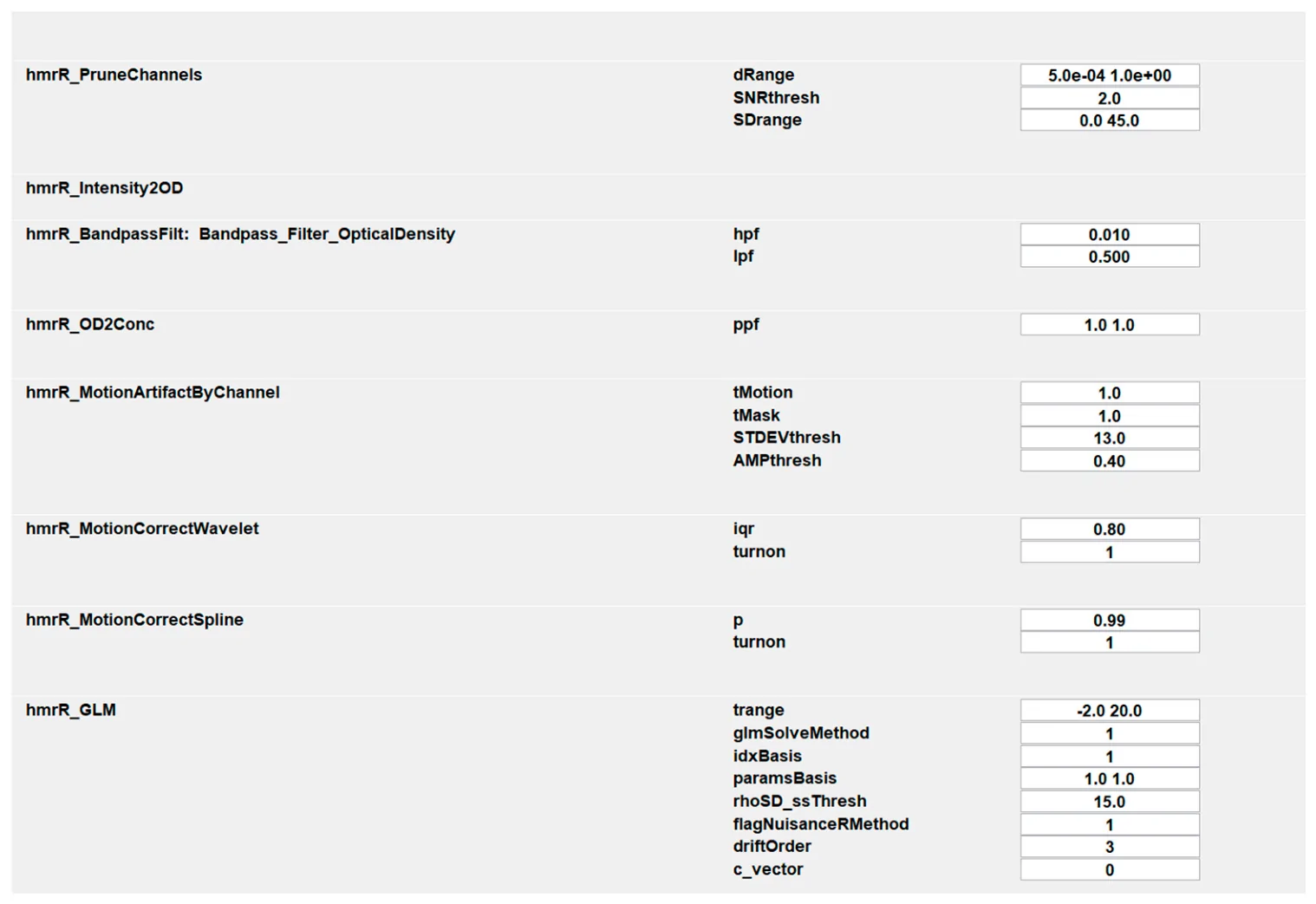
The proposed processing pipeline and respective thresholds in Homer3. The thresholds are either standard values in Homer3 or were determined based on previous autism studies using fNIRS [61,62].

The preliminary fNIRS data results, and the primary outcome of feasibility in this current paper, will be a simple assessment of the signal quality of the raw data (Table 2). Prior to data processing, this will involve a visual inspection of which channels yielded high-quality signals; i.e., channels with a visible and consistent cardiac pulsation, which is indicative of an appropriate hemodynamic response [60], and minimal visible noise due to movement or other artifacts. Then, data will be processed using Homer3 (v1.87.0) via MATLAB (R2024b), which will include pruning noisy signals, correcting the signal for motion artifact, converting the raw data to optical density, applying a bandpass filter to remove physiological noise, and converting to oxygenated hemoglobin concentration (HbO), deoxygenated hemoglobin concentration (HbR), and total hemoglobin concentration (HbT); see Figure 6 for the proposed Homer3 processing pipeline and thresholds for each respective step. These variables can then be used to infer neural activity in brain regions of interest for this task: the motor cortex, which is associated with movement, and the accessible area in the frontal cortex, which is associated with executive functions. These variables are listed in Table S1.

Once the data have been processed, we will assess feasibility based on percentage of usable channels. This will involve taking the number of channels remaining (i.e., those that have not been pruned or otherwise excluded after processing) for each participant, and dividing that number by the total number of channels (i.e., 20). Feasibility will be defined as a participant retaining 60% or more of the possible number of channels (i.e., ≥12 channels).

### 3.2. Motor Performance Results

The Meta Quest Pro Touch Controllers, independent of the Meta Quest Pro Headset itself, use a combination of embedded inertial measurement units (IMUs) and cameras to collect the x, y, and z positional coordinates of the participants’ hands in three-dimensional space. This raw 3D position data will be filtered at 4 Hz using a 2nd order Butterworth filter, a common preprocessing method in studies examining kinematic variables using similar VR systems [63,64]. Once filtered, we can calculate the following kinematic variables as preliminary measures of motor performance. The code, as currently written in Unity, accounts for tracking loss during movement. Any trials with recorded tracking loss will be excluded from analyses.

#### 3.2.1. Reaction Time

Reaction time (RT) is the time between stimulus presentation and movement initiation, measured in milliseconds (ms).

RT = Timestamp of Movement Initiation − Timestamp of Stimulus Presentation

To clarify, movement initiation is defined as the moment that the speed of the hand surpasses 0.01 m/s—a threshold that is commonly used to determine movement onset during reaching [62,65]. Accordingly, movement cessation is defined as the moment the speed of the hand is less than 0.01 m/s.

#### 3.2.2. Movement Time

Movement time (MT) is the time between movement initiation and movement cessation, i.e., the time elapsed between non-zero velocities throughout a movement—in this case, during reaching towards a target. MT will also be measured in milliseconds.

MT = Timestamp of Movement Cessation − Timestamp of Movement Initiation

#### 3.2.3. Time to Peak Velocity

Velocity is the change in position with respect to time, and will be calculated as the first derivative of the position of participants’ hands over the course of each reaching movement. It accounts for movement in both the horizontal and vertical direction, and is thus considered a vector. The time to peak velocity (TTPV), therefore, would be calculated as the total time elapsed between movement initiation and the peak velocity attained during the reach towards the target. TTPV will also be measured in milliseconds.

TTPV = Timestamp of Peak Velocity achievement − Timestamp of Movement Initiation

#### 3.2.4. Time to Peak Acceleration

Movement acceleration is the measure of how quickly an object speeds up or changes direction, and will be calculated as the second derivative of the position of participants’ hands over the course of each reaching movement. The time to peak acceleration (TTPA) would be calculated as the amount of time an object takes to reach maximum acceleration after initial movement. TTPA will also be measured in milliseconds.

TTPA = Timestamp of Peak Acceleration achievement − Timestamp of Movement Initiation

#### 3.2.5. Normalized Jerk

Normalized jerk (NJ), a dimensionless measure of variation in acceleration [66], quantifies the smoothness of a movement while accounting for the movement time and length [67]. Small quantities for jerk are indicative of maximum movement smoothness [66]. To calculate normalized jerk, we will use the formula used in Yan et al. (2000) [68], whose calculation methods were originally described by Kitazawa et al. (1993) [69] and Teulings et al. (1997) [70].

$$\mathrm{NJ}=\sqrt{(\frac{1}{2})\int j^{2}(t)*({duration}^{5}/{length}^{2})dt}$$

The above equation involves calculating jerk via the derivative of acceleration, then taking the square root of the time integral of squared jerk multiplied by the movement duratation^5^/length^2^.

## 4. Discussion

The above proposed methods have strong potential to yield a rich combination of kinematic, cognitive, and neural data. The use of neuroimaging during research-based tasks is crucial to expanding our understanding of autistic brain dynamics underlying cognitive–motor task performance. Furthermore, the use of neuroimaging and VR present individual and combined supplemental contributions to autism assessment methods. These assessments are invaluable and irreplaceable in the diagnostic sense—however, typical assessment and diagnostic measures, which rely on observational measures of behavior, can potentially be supplemented and augmented through the data yielded via technology such as fNIRS and VR. These methods also lay the groundwork for developing new, multifaceted interventions that can be tailored to individual support needs. VR, an already emerging means of intervention and support in autism research, has strong potential to address individual needs across the spectrum due to its inherently adaptable nature. The addition of neuroimaging provides more individualized data underlying task performance in the VR environment, offering the potential to tailor VR tasks to autistic brains across the spectrum.

We would be remiss to not mention the limitations of our design. Although fNIRS and VR have been individually well-tolerated in autistic youth, we must also consider the sensory aspect of combining these measures. It may be overwhelming to participants to have both apparatuses on their heads simultaneously—a hallmark of why beginning our work with feasibility assessments is crucial to move forward with the project as a whole. VR also poses the risk of cybersickness, which, like motion sickness, can cause symptoms such as nausea, disorientation, and visual discomfort. VR has been used previously in autism research with great success—nonetheless, our researchers will continuously check in with participants during the VR tasks and prioritize their comfort and well-being.

Furthermore, our current research-based task design can be considered limiting in and of itself. One task with various conditions/manipulations of cognitive and motor domains certainly cannot generalize to assessing cognitive and motor development at large, particularly considering the developmental heterogeneity among autistic youth. The behavioral and neural responses elicited will be specific to the task selected, and future work using additional tasks alongside this proposed task paradigm is needed for more comprehensive developmental assessment. Standardized assessments will also be crucial to this larger, holistic understanding of developmental domains in autism. These assessments, which are further detailed in the Supplementary Materials, target cognitive (e.g., Behavior Rating inventory of Executive Function—2nd edition (BRIEF-2)), motor (e.g., Bruininks-Oseretsky Test of Motor Proficiency—3rd edition (BOT-3)—Fine Motor Portion, and social and emotional development (e.g., Social Skills Improvement System (SSIS) Rating Scales). Once the feasibility of combining neuroimaging and virtual reality in autistic youth has been established, integrating the results of these standardized measures will afford the opportunity for validating our behavioral data, as well as allow us to examine the interrelationships between clinical profiles and cognitive and motor task performance.

This feasibility study and the preliminary cognitive and motor data gathered will set the groundwork for a multitude of future studies assessing the interrelationships between cognitive, motor, and social development in autistic children. Our preliminary, simplistic research-based task design sets the stage for creating much more complex tasks across various developmental domains, with VR affording malleable, controllable, and extremely realistic environments. We hope by combining neuroimaging and virtual reality paradigms, we can use these methods in addition to traditional assessments, such as the ADOS. Furthermore, in the long-term, this work will advance multi-modal, holistic, and comprehensive autism research—viewing these relationships and their neural correlates through a multifaceted lens will inform our understanding of the autistic brain, diversify autism assessment methods, and improve intervention strategies across the autism spectrum.

## Figures and Tables

**Table 1:** Research-based task descriptions and cognitive and motor outcome measures.

VR Task: Developmental Domain	Task Description	Brain Region(s) of Interest	Outcome Variables
Cognitive	Go/No-Go task: A Go/No-go paradigm in which participants must “pop” a series of target items—purple balloons—while leaving all blue balloons alone. Balloons will be presented one at a time in randomized locations. Balloon popping will be achieved by a button press on the handheld controller associated with the VR headset (i.e., participants’ hands will remain stationary/in their lap).	Prefrontal cortex Dorsolateral prefrontal cortex	Primary (feasibility-based): Commission Errors (responding to a “no-go” stimulus) fNIRS signal quality
Motor	Reach-and-Pop Balloon Task: Modified version of bubble-popping task in Minissi et al., 2023 [34]; balloons of one color will be presented one at a time at randomized locations and must be popped. The player must reach towards the balloon’s location and then pop the balloon using the button press on the handheld controller. After each balloon, participants must reset their hand to a predefined starting position (under their chin). This will activate the next balloon’s appearance.	Motor Cortex	Primary (feasibility-based): Reaction Time Movement Time Time to Peak Velocity Time to Peak Acceleration Normalized Jerk fNIRS signal quality
Cognitive–Motor	Go/No-Go Reach-and-Pop Task: A combination of the above cognitive and motor tasks, i.e., a Go/No-go Reach-and-Pop paradigm, in which participants must discern between differently colored balloons to reach and pop all purple balloons while blue balloons must be left alone.	Prefrontal cortex Dorsolateral PFC Motor cortex	Primary (feasibility-based): Commission Errors (responding to a “no-go” stimulus) Reaction Time Movement Time Time to Peak Velocity Time to Peak Acceleration Normalized Jerk

**Table 2:** Primary procedures and outcome measures: feasibility assessment and preliminary behavioral and neural outcomes of research-based tasks.

Consented Individual Involved	Procedure/Assessment	Outcome Measures
Child	Subjective Feasibility Assessment	Tolerability ○ “Was the VR headset comfortable to wear?” ○ “Was the brain cap comfortable to wear?” ○ “Did the tasks make you tired?” ○ “Did you feel capable of completing these tasks?” ○ “Did the tasks feel too challenging?” Enjoyability ○ “Did you enjoy these tasks?” ○ “Would you do them again?” ○ “Were the tasks engaging?”
N/A (To be completed by researchers after participant’s study visit.)	Objective Feasibility Assessment	How long did the entire testing battery take? How many participants completed all assessments and tasks? Preliminary behavioral and neural data—how was the data quality? Are the findings reflective of previous literature?
Child	Research-Based Tasks: Select Neural Outcome Measures	fNIRS data quality (percent of channels yielding usable, high-quality signals; ≧60% of the total possible number of channels)
Child	Research-Based Tasks: Select Behavioral Outcome Measures	Motor Outcome Measures ○ Reaction Time ○ Movement Time ○ Time to Peak Velocity ○ Time to Peak Acceleration ○ Normalized Jerk Cognitive Outcome Measures ○ Commission Errors (responding to a “no-go” stimulus)

## Data Availability

No new data were created or analyzed in this study. Data sharing is not applicable to this article.

## Supplementary Materials

The following supporting information can be downloaded at: https://www.mdpi.com/article/10.3390/brainsci16090947/s1, Table S1: Secondary, Exploratory Procedures and Outcome Measures: Exploratory cognitive and neural outcomes of research-based tasks; Table S2: Tertiary, Exploratory Procedures: Demographics, clinical descriptives, and other sample characterizations.

## Abbreviations

The following abbreviations are used in this manuscript:

ADOS-2	Autism Diagnostic Observation Schedule—2nd edition
ADI-R	Autism Diagnostic Interview-Revised
fMRI	Functional Magnetic Resonance Imaging
fNIRS	Functional Near-Infrared Spectroscopy
HbO	Hemoglobin Concentration
HbR	Deoxygenated Hemoglobin Concentration
HbT	Total Hemoglobin Concentration
IMUs	Inertial Measurement Units
LCD	Liquid Crystal Display
MEG	Magnetoencephalography
MS	Milliseconds
MT	Movement Time
NJ	Normalized Jerk
SSQ	Simulation Sickness Questionnaire
RT	Reaction Time
TTPV	Time To Peak Velocity
TTPA	Time To Peak Acceleration
VR	Virtual Reality
