# Supplementary material for: Cognitive and Motor Development in Autistic Children: A Multi-Modal Feasibility Study Combining Neuroimaging and Virtual Reality Paradigms
Source: Brain Sci. 2026 Sep 5;16(9):947. doi: 10.3390/brainsci16090947 (PMC13604929; doi:10.3390/brainsci16090947)
Supplement: Supplementary file 1 [file brainsci-16-00947-s001.zip › brainsci-4471546-supplementary.pdf]

Table S1. Secondary, Exploratory Procedures & Outcome Measures: Exploratory cognitive and neural outcomes of research-based tasks.

Consented Individual Involved	Procedure/Assessment	Outcome Measures
Child	Research-Based Tasks: Exploratory Neural Outcomes Measures	fNIRS: HbO, HbR, HbT concentrations
Child	Research-Based Tasks: Exploratory Behavioral Outcomes Measures	*Additional Cognitive Outcomes Across Cognitive and Cognitive-Motor Conditions*<br>• Accuracy (percentage of correct responses on both "go" and "no-go" trials)<br>• False Alarm Rate (percentage of "no-go" trials where a response was made<br>• Commission Errors (responding to a "no-go" stimulus)<br>• Omission Errors (failing to respond to a "go" stimulus)

Table S2. Tertiary, Exploratory Procedures: Demographics, clinical descriptives, and other sample characterizations.

Consented Individual Involved	Procedure/Assessment
Child	Autistic Diagnosis Observation Schedule – 2nd Edition (ADOS-2)

Child	The Wechsler Abbreviated Scale of Intelligence – 2nd Edition (WASI-II)
Child	Bruininks-Oseretsky Test of Motor Proficiency – 3rd Edition (BOT-3) – *Fine Motor Portion*
Child	Screen for Anxiety Related Child Disorders (SCARED)
Child	UCLA Loneliness Measure (modified)
Child	fNIRS Qualitative Questions
Parent	Demographics and Diagnostic History
Parent	Social Communication Questionnaire (SCQ)
Parent	The Social Responsiveness Scale – Second Edition (SRS-2)

Parent	Child Behavior Checklist (CBCL)
Parent	Behavior Rating inventory of Executive Function – 2nd edition (BRIEF-2)
Parent	Social Skills Improvement System (SSIS) Rating Scales
Parent	The Child and Adolescent Scale of Participation (CASP) Parent Report
Parent	Stanford Social Dimensions Scale
Parent	Movement Assessment Battery for Children, Third Edition (MABC-3) Checklist
